# An interactive, self-guided tutorial on scientific writing for first year physiology students

**DOI:** 10.1016/j.crphys.2025.100157

**Published:** 2025-07-23

**Authors:** Alex Swainson, Matthew J. Mason, Frances M. MacMillan

**Affiliations:** a University of Bristol, School of Physiology, Pharmacology and Neuroscience, Bristol, BS8 1TD, United Kingdom; b University of Cambridge, Department of Physiology, Development & Neuroscience, Downing Street, Cambridge, CB2 3EG, United Kingdom

## Abstract

Most biomedical science students arriving at UK universities have very limited experience of writing scientifically and have little insight into the process involved in producing a peer-reviewed academic publication. To help support them, we created an interactive, online tutorial to help improve their scientific writing through looking at aspects including the construction of a logical argument, use of figures and referencing, as well as providing an overview of the publication process. The tutorial was delivered in an in-person teaching workshop at the University of Bristol and offered as an optional, online-only activity at the University of Cambridge, in both cases to first-year physiology students. In Bristol, 68 % of 152 students and in Cambridge, 67 % of 561 students engaged with the interactive tutorial. These students were invited to complete before-and-after surveys, with questions relating to their confidence in and understanding of the topics covered. Feedback from students in both institutions was overwhelmingly positive, with a statistically significant increase in reported confidence and understanding following completion of the tutorial. We propose the use of similar interactive tutorials as a simple, low-investment way in which training in scientific writing can be included in undergraduate science curricula, to help students prepare for what is expected in coursework, exam essays and in their postgraduate careers.

## Highlights

• Interactive online H5P tutorial to support Biomedical Science students' scientific writing skills, with a focus on essays and the publication process.
• Tutorial was delivered in-person or online at Universities of Bristol and Cambridge. Student feedback was collected via pre- and post-questionnaires.
• Student confidence and understanding increased significantly, supporting use of the tutorial to scaffold undergraduate scientific writing training.

## 1 Introduction

Writing skills are integral to scientific degree schemes and indeed, to wider scientific careers. From journal articles and reviews to grant applications, we rely on written communication to generate and disseminate research within the academic community and beyond. However, despite many guides to academic writing, we often see confusion, lack of confidence and even fear from undergraduate students regarding their scientific writing skills (Reynolds et al., 2011; Turbek et al., 2016; French, 2018; Grogan, 2021). Essays in particular, while viewed by many academics as a useful tool for the development of students’ writing and critical thinking skills, are often met with anxiety by the students themselves (French, 2018; Lavelle et al., 2013; Martinez et al., 2011; Dahl et al., 2023). Exacerbating this, while many marking schemes identify critical analysis and well-composed arguments as skills necessary for high grades, teaching often focuses on the scientific concepts, rather than the skills required to communicate them (Turbek et al., 2016). In some instances, students are expected to act on vague or unclear feedback (Chanock, 2000), or to identify, understand and employ the scientific writing style indirectly by reading academic journals (French, 2018; Joshi, 2007). These challenges may be intensified for students from educationally disadvantaged backgrounds, and for students whose first language is not English (French, 2018; Pineteh, 2013).

Another challenge currently faced by universities is to create programmes for a changing world. This requires, but is not limited to, the creation of flexible, blended modes of delivery to meet students’ needs. The UK QAA benchmark for Biosciences (2023) states that “learning and teaching strategies in the Biosciences are not static but adapt to changes in philosophy and technology in innovative ways that are accessible and inclusive to all” (QAA, 2023). In line with this, the creation and integration of interactive tutorials enables students to complete a programme of instruction remotely, return to the resource for revision or when competing coursework, and also means that students can break the work down into smaller, more approachable segments in a self-paced manner, as required. Interactive resources are also often screen reader-compatible, and may increase engagement and interactivity within a unit, as well as providing active learning opportunities (University of Plymouth, 2022). They are often well received by undergraduate students, who have reported a desire for more interactive elements in their courses (Jacob et al., 2024).

At the University of Bristol, we previously ran large-group lectures to introduce first-year physiology students to scientific essay writing (Naish et al., 2015; MacMillan et al., 2017). This included information on the peer review process and the evaluation of example essays, to support students in their production of formative and summative coursework essays. The lecture format provided limited opportunities for interaction and engagement and the students were unable to revisit the material later. To overcome these challenges, we created an interactive tutorial that includes much of the same information, but which forms a more active, student-centred activity that can be completed in-person, asynchronously, in groups or individually, allowing students with different experience and needs to work through the information in a self-paced manner that works best for them. What was presented as a PowerPoint presentation in the original lecture was converted to an interactive tutorial created using H5P. This tutorial covers the production of scientific essays, as well as introducing students to the scientific publication process (see Methods for more details). For each activity, students receive feedback on their performance in real-time.

The online nature of this tutorial is especially beneficial for courses in which there is limited space within the timetable for an in-person, synchronous workshop on scientific writing, or for which staffing or logistics would not allow for such an activity. In the University of Cambridge, science students write formative essays set and marked by individual tutors (‘supervisors’), with very little cohort-wide training provided on scientific writing. We introduced our essay-writing tutorial as an optional, online activity for first-year undergraduate students enrolled on the Natural Science, Medicine and Veterinary Medicine courses. In this paper, we report on the outcome of introducing our interactive tutorial within both Bristol and Cambridge learning contexts, based on the results of feedback surveys completed by students who engaged in this exercise.

## 2 Methods

### 2.1 The interactive tutorial

**Fig. 1:**
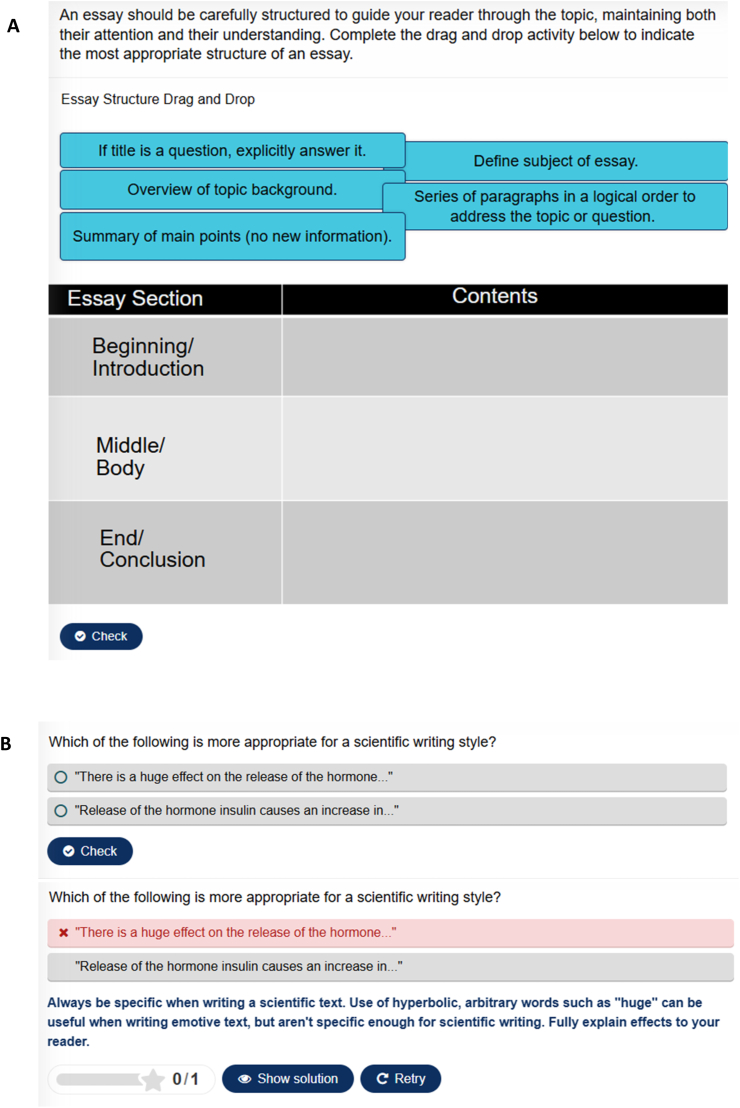
**Example Tutorial Activities**. A) A drag-and-drop exercise outlining the content expected in the start, middle and end of scientific essays. B) A multiple-choice question on scientific writing style, with feedback provided.

The interactive tutorial was written within the HTML5 Package (H5P) and run online. It is comprised of a series of ’pages', each covering one aspect of essay-writing practice. The tutorial begins with 5 pages covering scientific writing in a broad sense, and the process involved in publishing a journal article, followed by 11 pages covering scientific writing style and essay structure, including how to create a logical and well-supported argument. 3 pages cover the use of figures and equations, and a further 5 provide information on plagiarism, referencing, the use of online resources and AI. Within the tutorial, students complete activities to support and reinforce the information presented. These activities include drag-and-drop, answering multiple-choice questions and rearranging sentences (Fig. 1; for additional examples of activities, please see the ‘H5P examples’ PDF in the Open Science Framework project linked below). The tutorial provides instant feedback on the activities that students can revisit when completing coursework or revising for exams. An exercise at the end of the tutorial allows students themselves to mark sample essays, according to given criteria. We estimate that the tutorial would normally take a student 1–2 h to complete.

### 2.2 Participants and questionnaires

Participants from the University of Bristol included first-year biomedical students taking the Physiology 1A unit, which includes students enrolled on Physiological Science, Neuroscience, Pharmacology and Applied Anatomy BSc and MSci courses. These students were expected to complete the tutorial within an essay-writing workshop (see later), designed to support their later completion of formative and summative coursework essays.

Participants from the University of Cambridge included first-year undergraduate students enrolled in the natural sciences, medical and veterinary programmes, as well as students enrolled in the graduate medicine programme who also take the first-year medical physiology course. The undergraduates write formative essays regularly throughout the year as part of their small-group teaching sessions and take an essay paper as part of their end-of-year summative examinations. Graduate medical students are not assessed by essay. In Cambridge, the essay-writing tutorial and associated questionnaires were available online only.

The pre- and post-tutorial questionnaires, which were the same in both universities, included six Likert-scale questions and a box for free text comments. It was explained that completing these anonymous questionnaires was completely optional, and those who chose not to would still be able to complete the essay-writing tutorial with no negative consequences. In Bristol, participants’ names were recorded for the purposes of matching the pre- and post-tutorial questionnaire answers, but all were anonymised prior to analysis. In Cambridge, students were asked to generate a pseudonym to link their two questionnaires anonymously. Ethical approval was obtained from Faculties of Life Sciences and Science Research Ethics Committee in Bristol (approval code: 15999) and the Cambridge Higher Education Studies Research Ethics Committee (approval code 2024.ET.72.Mason).

The six Likert-scale questions (with possible answers ranging from 1 not at all - 5 yes, very well) asked in both pre- and post-tutorial questionnaires were as follows:

1) I understand what is meant by a scientific writing style.
2) I feel confident writing essays using a scientific writing style.
3) I understand the basic structure of an essay, and what information each section should contain.
4) I feel confident in writing and applying essay plans in my essay writing.
5) I feel confident in assessing the quality of my own essay writing prior to submission.
6) I understand the publishing process for scientific journal articles.

The questions were designed to assess the students' understanding and confidence in the writing process as a whole, rather than individual elements of the tutorial such as the production of figures.

### 2.3 Presentation of the tutorial

In Bristol, students completed the H5P tutorial either individually or as groups, within a facilitated, 2-h, in-person workshop which took place in the second week of teaching, in term 1. The group was asked to pause at regular intervals throughout the tutorial to reflect on each section through comments posted on Padlet. The workshop was held in a flat-bed teaching space, with staff answering questions and clarifying understanding as needed.

In Cambridge, the H5P tutorial was slightly adapted from the Bristol version to fit local requirements and embedded into Moodle, the virtual learning platform used in the physiology courses. The self-guided tutorial was available from the start of the academic year, with no fixed completion date, and was completed asynchronously with no staff facilitation. Students were encouraged to take the tutorial by the course organisers but student participation was neither compulsory nor individually monitored. No time was set aside in the course to take the tutorial, but some of the students’ academic supervisors, who independently run weekly small-group teaching sessions, may have asked their own students to complete the tutorial for homework. The vast majority of those who took the tutorial (94 %) did so within the first month of their first term.

### 2.4 Statistical analyses

Quantitative data collected from students who completed both the pre- and post-tutorial questionnaires were analysed using paired Student's t-tests: the use of parametric tests in analysing Likert-scale data is defended by Norman (2010). General linear models were used to compare data between institutions. Qualitative data arising from students' additional comments were analysed via thematic analysis.

## 3 Results

### 3.1 Participation

In Bristol, of the 152 registered students, 103 attended the workshop. Of these, 64 completed both pre- and post-tutorial questionnaires. In Cambridge, of the 561 students registered across the three programmes, 376 logged into the tutorial and of these 123 completed both the pre- and post-tutorial questionnaires. Therefore, 68 % and 67 % of students in Bristol and Cambridge respectively engaged with the interactive tutorial in some way, although we cannot be sure that all of these students actually completed it.

### 3.2 Questionnaire scores

**Fig. 2:**
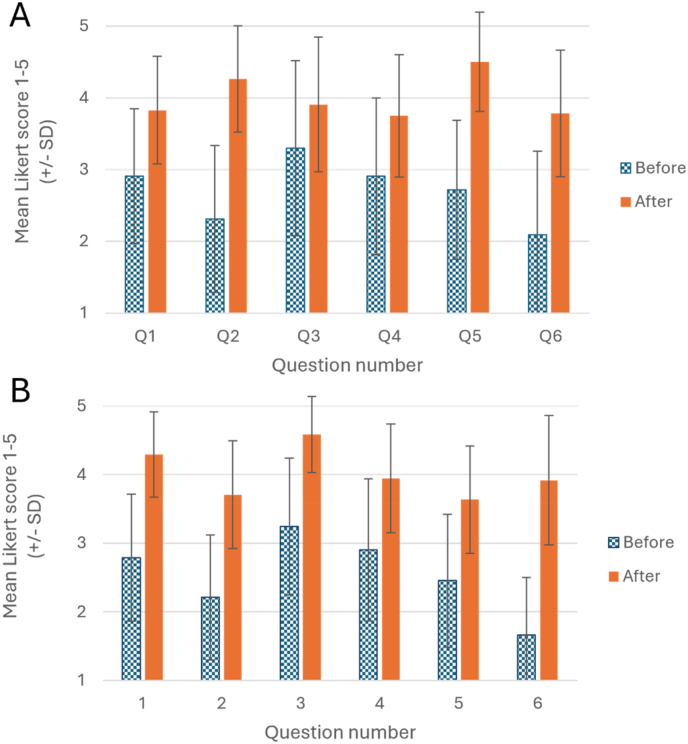
**Mean feedback scores from students before and after completing the essay-writing tutorial.** A) Mean pre- and post-tutorial (‘before and after’) scores from students at the University of Bristol. B) Mean before and after scores from students at the University of Cambridge. Across both institutions, scores for all questions increased significantly after completion of the essay writing workshop (p < 0.001).

The mean Likert-scale questionnaire scores collected from students at both universities for all questions 1–6 increased significantly when comparing pre- and post-tutorial answers using paired t-tests (Table 1; Fig. 2).

**Table 1:** Comparison of pre- and post-tutorial Likert scores for questions 1–6, assessed by paired *t*-test (t = test statistic, df = degrees of freedom).

	Question	Bristol			Cambridge		
		t	df	p	t	df	p
Q1	I understand what is meant by a scientific writing style	6.97	63	<0.001	19.34	122	<0.001
Q2	I feel confident writing essays using a scientific writing style	14.73	63	<0.001	19.45	122	<0.001
Q3	I understand the basic structure of an essay, and what information each section should contain	3.43	63	<0.001	15.69	122	<0.001
Q4	I feel confident in writing and applying essay plans in my essay writing	6.05	63	<0.001	12	122	<0.001
Q5	I feel confident in assessing the quality of my own essay writing prior to submission	14.04	63	<0.001	14.58	122	<0.001
Q6	I understand the publishing process for scientific journal articles	10.14	63	<0.001	23.22	122	<0.001

### 3.3 Comparison of data between institutions

#### 3.3.1 Pre-tutorial likert scores

**Fig. 3:**
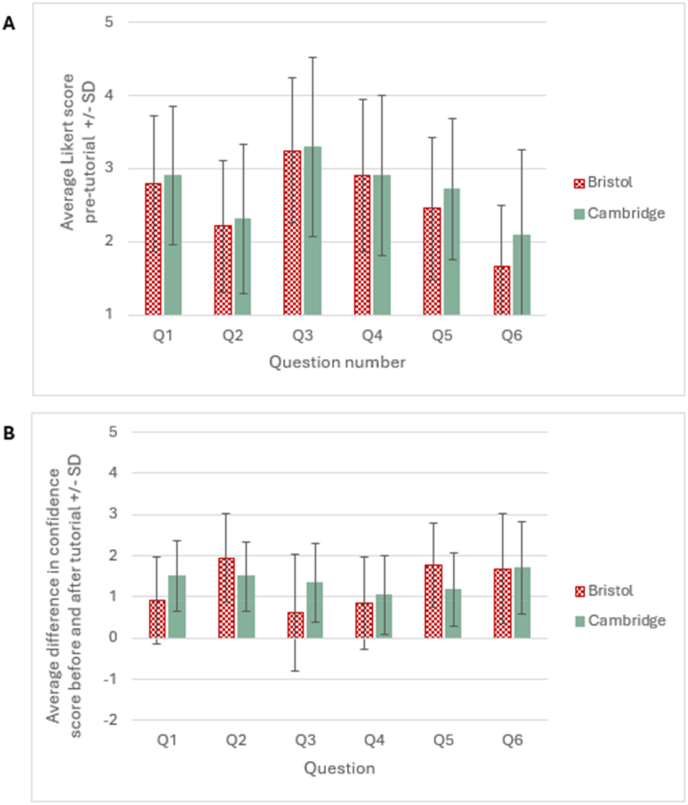
**Comparison of data between the Universities of Bristol and Cambridge.** A) Pre-tutorial scores. Scores did not differ significantly between students completing the tutorial in Bristol and Cambridge, but did differ between questions. B) Comparison of the difference in scores from students before and after completing the essay-writing tutorial, in Bristol and Cambridge. This did not differ significantly between students completing the tutorial in Bristol and Cambridge, but did differ between questions.

A general linear mixed model (GLM) was built to identify any differences in the pre-tutorial Likert scores between institutions studied (n = 187). Covariates included question number and institution, and participant pseudonym was used as a random factor, to control for repeated measures. Unsurprisingly, results indicated that pre-tutorial scores differed between the questions asked (F(5, 930) = 91.7, p = <0.001). Results of a Tukey HSD post-hoc test indicated that Likert scores differed significantly across all questions (p = <0.001) with the exception of questions 1 and 4 (p = 0.924), indicating that students’ scores differed across the competencies surveyed. However, pre-tutorial scores did not differ between institutions (F(1, 185) = 2.05, p = 0.15; Fig. 3a).

#### 3.3.2 Differences in likert scores pre- and post-tutorial

A second GLM was built to identify any differences in how the pre-to post-tutorial scores changed, between institutions (n = 187). Differences in scores were calculated for all students pre-to post-tutorial, and covariates included question number and institution. Again, participant pseudonym was used as a random factor, to control for repeated measures. Results suggested that the difference in pre-to post-tutorial scores differed between questions (F(5, 930) = 35.76, p = <0.001). A Tukey HSD post-hoc test suggested that difference scores for all questions differed significantly (P = <0.05), with the exceptions of Q1 and Q3 (P = 0.2), Q1 and Q5 (p = 0.96), and Q3 and Q4 (p = 0.081). This indicates that students felt the tutorial helped to different extents, depending on the question asked. However, there was no difference in the pre-to-post tutorial Likert scores between institutions (F(1, 185) = 2.84, p = 0.09; Fig. 3b).

### 3.4 Open-ended comments

#### 3.4.1 Bristol

**Fig. 4:**
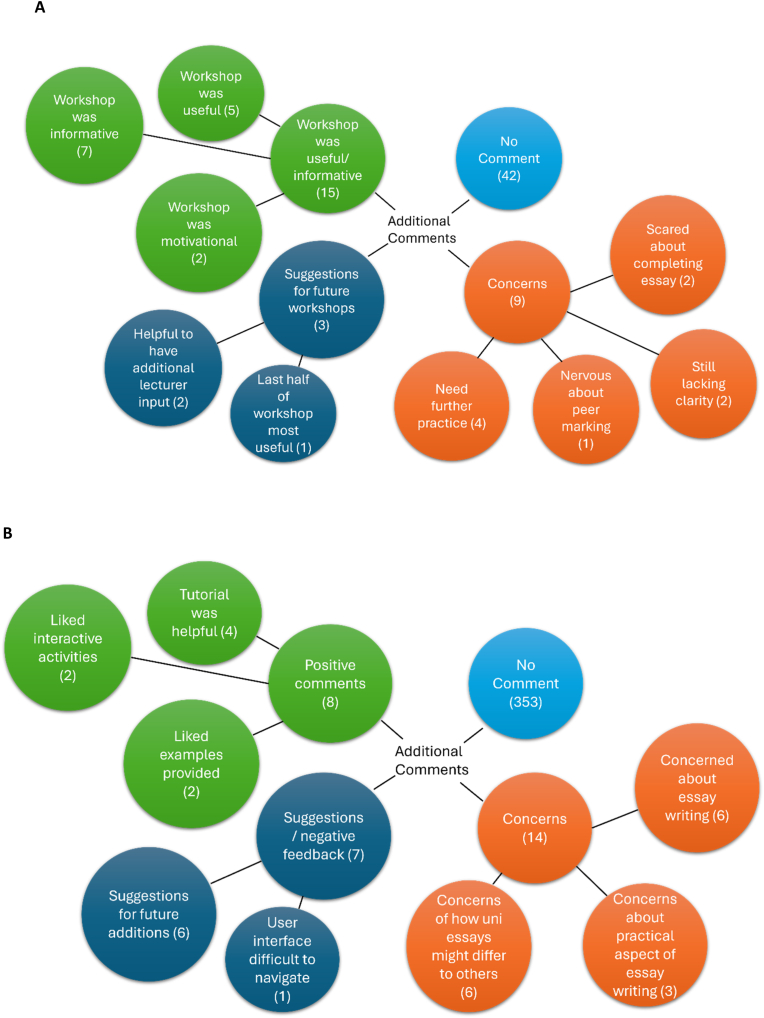
**Additional comments provided by students, clustered into four main themes.** A) Comments from students at Bristol, number of comments in brackets. B) Comments from students at Cambridge, number of comments in brackets. Some longer comments included more than one point, coded separately. Comments containing positive statements are coded in green, comments that reflect student concerns are coded in orange, comments that provided suggestions and feedback on the tutorial itself are coded in dark blue. The number of students that provided no additional comments is outlined in light blue.

Thematic analysis identified four general themes arising from students’ additional comments at the University of Bristol (Fig. 4a). While 42 out of 64 Bristol respondents provided no additional comments, 14 indicated that the workshop was useful and informative, particularly ahead of the required coursework for the unit. Example comments include:

“Much more sound understanding of scientific essays in general and how they are marked”

“The examples of essays were quite useful”.

However, 4 students commented that they were still confused regarding the scientific writing style, and an additional 4 reported apprehension ahead of the associated essay coursework and peer marking, despite completion of the workshop. This was perhaps because students did not write an essay themselves in the workshop:

“Overall, it was an insightful workshop. Though it was mostly theoretical and I haven’t tried it out practically, so it’s a bit difficult to assess properly of my understanding in a sense that I could actually put it into practice. Hopefully will be able to do that in my own time later on”.

The final theme related to suggestions for workshop improvement going forward. Two students commented that the workshop would benefit from additional lecturer input:

“I think the workshop would be more helpful if some of it was presented to us by a lecturer, just to clarify some of the key points”.

#### 3.4.2 Cambridge

Of the 376 students who logged into the tutorial, only 23 left open-ended comments. No student provided comments both pre- and post-tutorial. Thematic analysis of comments once again identified four general themes arising from the data (Fig. 4b). 8 positive comments were left regarding the tutorial of which 4 students commented that the tutorial was helpful, there were 2 positive comments on the interactive nature of the workshop, and a further 2 positive comments related to the example essays included. Example comments include:

“Overall, I found this course really helpful in understanding more about writing scientific essays at university level. Thank you.”

“Thank you so much!! It was really helpful.”

The third theme centred around student's concerns regarding essay writing, and the 14 responses in this vein were almost exclusively collected from pre-tutorial responses. 6 students commented that they were worried about scientific essay writing in general, and 6 students were concerned about how essays differ between subjects, or between school and university. 3 students left comments outlining concerns around the practical aspects of essay writing, such as referencing (1), getting an essay to flow (1), using diagrams and tailoring essays to titles (1). Examples include:

“I have had some experience writing scientific essays, but I am unsure on how the jump to uni-level essays is going to be.”

“I find the hardest part of writing an essay is starting the actual essay. I can think of ideas but coming with a good introduction and making the whole essay flow nicely is something I struggle with.”

“I would very much like to include diagrams, but am still unsure how best to label these clearly so they support but do not stand in for the text-based content of my essay.”

The final theme included comments on areas for improvement on future iterations of the tutorial. 6 comments provided suggestions for additional content, and 1 comment referred to difficulties interacting with the user interface. Examples included:

“I liked the example essays that have been included and the interactive activity of assembling into the most logical order, could have more detail about how to construct an argument and structure such an argument into paragraphs (ensuring a logical flow).”

“The user interface on the drag and drop exercises was quite poor, forcing me to pick up and drop tiles repeatedly to get them down.”

All comments can be found in the Open Science Framework project linked below.

## 4 Discussion

Although both Bristol and Cambridge are Russell-group universities with highly selective admissions processes, responding students from both institutions reported a strikingly low level of confidence in writing essays in a scientific style, prior to the tutorial (Question 2; Fig. 3A). The improvement in confidence after taking the tutorial (Fig. 2) was gratifying, and a key aim of the resource during its development. Whilst it may be tempting to correlate student engagement with the tutorial with their subsequent essay grades, we had no way of knowing whether the cohorts which engaged with the tutorial were disproportionately made up of the most committed students, or those who had the least experience with scientific essay-writing and felt they needed the most help, making any relationship difficult to interpret. Furthermore, the marking of summative essays in both universities does not focus solely on the quality of scientific writing but also depends upon the scientific content of their essays. As such, the tutorial was designed more as a general introduction to the scientific writing style and a reference tool for students to use throughout their degrees. Perhaps unsurprisingly, Likert scores differed significantly across all questions asked, with Question 6 receiving particularly low scores. This is presumably due to undergraduate students having little previous experience of the scientific publication process. We believe that linking essay-writing with scientific publishing is important in helping physiology students to see their writing exercises as a transferrable skill which may aid them in the future.

Interestingly, similar results were seen across both institutions, despite differences in the modes of delivery of the tutorial. Students across both institutions returned similar Likert scores before completing the tutorial, suggesting that neither cohort was more assured of their writing skills than the other. The low number of graduate students completing the tutorial from Cambridge (just two students identified as anything other than an undergraduate despite there being 30 students on the graduate medicine course) perhaps reflects the fact that graduates have prior experience in essay writing, or that they are not required to write summative essays as part of their end-of-year examinations. At the University of Bristol, the facilitated workshop was held in a flatbed teaching space, a type of environment believed to improve engagement by reducing the distance - both physical and relational - between students and teachers, to foster informal and inclusive discussions (Oblinger, 2006; Acton et al., 2020). By holding the workshop in this space, we aimed to promote collaboration between staff and students, and students with their peers, in order to break down the hierarchical barrier between staff and students that traditional lecture theatres can create (Oblinger, 2006). Flat-bed teaching environments are often especially beneficial for neurodiverse students, perhaps because the relaxed and informal atmosphere gives such students “permission” to be themselves, improving their learning experience (Marche, 2022). However, the self-reported benefits were similar between students exposed to the tutorial in a classroom format in Bristol and online-only in Cambridge. The results of this study therefore suggest that the format of delivery does not have a significant effect on the perceived benefits. As such, institutions can select the format that would work best for their students, staff and timetabling constraints.

The interactive tutorial created using H5P provides, instant feedback, setting a clear standard to all students, regardless of skill level, previous experience or background (French, 2018). Following the successful introduction of the interactive tutorial across both institutions, we plan to continue its use in years to come. While attendance figures appear relatively low for students at Bristol, this was likely due to the introduction of a new check-in app, which was subject to technical problems at the time the workshop was held. The tutorial was felt to work well as an asynchronous, optional resource in Cambridge, and will continue in that format. Future editions will accommodate students’ feedback suggestions where possible, adding sections and examples where appropriate, and we will enhance lecturer input to the facilitated in-person workshop in Bristol. Some students were disappointed that the tutorial did not include the opportunity to write their own essays to put into practice what they had learned. This was not offered due to both time constraints and the position of the Bristol workshop in the academic year. However, later in their respective courses, students do get the opportunity to write their own essays, for which they receive individual formative feedback.

It should be noted that essay–writing has been subject to scrutiny in higher education, in comparison to more ‘authentic’ methods of assessment and in the light of concerns about academic integrity and student use of AI (Macandrew et al., 2002; Lapworth, 2021). The QAA Benchmark for Biosciences (2023) suggests that authentic methods of assessment in academic writing might include scientific communications aimed at a range of audiences, including posters, graphical resources, videos and websites, as well as more traditional publications such as journal articles, scientific reports and reflective writing portfolios. Although our tutorial ostensibly focuses on essay-writing, the increased awareness and confidence in scientific writing that it offers will be transferrable to many different forms of communication which our students might be asked to contribute to in the future.

We acknowledge that there is not just one way to write a scientific essay. Some academics will certainly disagree with some of the specific recommendations in our tutorial or may be concerned that students will believe that there is a particular formula that they must follow. In response, we would point to the low levels of confidence of our students in essay-writing before taking the tutorial, which suggests that some form of support and guidance is needed at the start of their university careers. Our tutorial is no substitute for personalised formative feedback on essays written by the students themselves, as part of which the advantages of alternative styles and essay structures can and should be discussed. However, we believe that the increased confidence in scientific writing shown by students who took our tutorial gives them the scaffolding on which to begin such conversations.

## CRediT statements

Swainson et al.

Conceptualization: AS, FMM.

Methodology: AS, FMM, MJM.

Formal analysis: AS.

Investigation: AS, FMM, MJM.

Resources: AS, FMM, MJM.

Data Curation: AS, MJM.

Writing - Original Draft: AS, FMM, MJM.

Writing - Review & Editing: AS, FMM, MJM.

Supervision: FMM.

Project administration: AS, FMM, MJM.

## Declaration of competing interest

The authors declare that they have no known competing financial interests or personal relationships that could have appeared to influence the work reported in this paper.

## Data Availability

Data will be made available on request.
